# The Apolipoprotein E neutralizing antibody inhibits SARS‐CoV‐2 infection by blocking cellular entry of lipoviral particles

**DOI:** 10.1002/mco2.400

**Published:** 2023-10-10

**Authors:** Qi Cui, Arjit Vijey Jeyachandran, Gustavo Garcia, Chao Qin, Yu Zhou, Mingzi Zhang, Cheng Wang, Guihua Sun, Wei Liu, Tao Zhou, Lizhao Feng, Chance Palmer, Zhuo Li, Adam Aziz, Brigitte N. Gomperts, Pinghui Feng, Vaithilingaraja Arumugaswami, Yanhong Shi

**Affiliations:** ^1^ Department of Neurodegenerative Diseases Beckman Research Institute of City of Hope Duarte California USA; ^2^ Department of Molecular and Medical Pharmacology UCLA Los Angeles California USA; ^3^ Section of Infection and Immunity Herman Ostrow School of Dentistry Norris Comprehensive Cancer Center University of Southern California Los Angeles California USA; ^4^ Electron Microscopy and Atomic Force Microscopy Core Beckman Research Institute of City of Hope Duarte California USA; ^5^ Mattel Children's Hospital UCLA Department of Pediatrics David Geffen School of Medicine UCLA UCLA Children's Discovery and Innovation Institute Los Angeles California USA; ^6^ UCLA Molecular Biology Institute Los Angeles California USA; ^7^ UCLA Jonsson Comprehensive Cancer Center Los Angeles California USA; ^8^ UCLA Eli and Edythe Broad Stem Cell Research Center Los Angeles California USA; ^9^ Division of Pulmonary and Critical Care Medicine Department of Medicine UCLA David Geffen School of Medicine Los Angeles California USA

**Keywords:** antiviral therapy for SARS‐CoV‐2, ApoE, ApoE neutralizing antibody, ApoE receptors, human iPSC‐derived astrocytes, LDLR

## Abstract

Severe acute respiratory syndrome coronavirus 2 (SARS‐CoV‐2) is the causal agent for coronavirus disease 2019 (COVID‐19). Although vaccines have helped to prevent uncontrolled viral spreading, our understanding of the fundamental biology of SARS‐CoV‐2 infection remains insufficient, which hinders effective therapeutic development. Here, we found that Apolipoprotein E (ApoE), a lipid binding protein, is hijacked by SARS‐CoV‐2 for infection. Preincubation of SARS‐CoV‐2 with a neutralizing antibody specific to ApoE led to inhibition of SARS‐CoV‐2 infection. The ApoE neutralizing antibody efficiently blocked SARS‐CoV‐2 infection of human iPSC‐derived astrocytes and air–liquid interface organoid models in addition to human ACE2‐expressing HEK293T cells and Calu‐3 lung cells. ApoE mediates SARS‐CoV‐2 entry through binding to its cellular receptors such as the low density lipoprotein receptor (LDLR). LDLR knockout or ApoE mutations at the receptor binding domain or an ApoE mimetic peptide reduced SARS‐CoV‐2 infection. Furthermore, we detected strong membrane LDLR expression on SARS‐CoV‐2 Spike‐positive cells in human lung tissues, whereas no or low ACE2 expression was detected. This study provides a new paradigm for SARS‐CoV‐2 cellular entry through binding of ApoE on the lipoviral particles to host cell receptor(s). Moreover, this study suggests that ApoE neutralizing antibodies are promising antiviral therapies for COVID‐19 by blocking entry of both parental virus and variants of concern.

## Graphical abstract

How SARS‐CoV‐2 virus infects cells that express no or low levels of human ACE2 remains to be explored. This study shows that SARS‐CoV‐2 can hijack the lipid‐binding protein ApoE to be part of the lipoviral particle. SARS‐CoV‐2 can enter host cells through the interaction of ApoE on the lipoviral particles and ApoE receptor(s) on host cell surface. An ApoE neutralizing antibody can efficiently inhibit the infection of SARS‐CoV‐2 and its variants of concern.

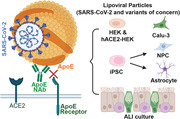

## 1 INTRODUCTION

The coronavirus disease 2019 (COVID‐19) pandemic has presented a massive global public health threat. The severe acute respiratory syndrome coronavirus 2 (SARS‐CoV‐2), a positive‐strand RNA virus, is the causal agent for this pandemic. SARS‐CoV‐2 and its variants/subvariants emerged after the parental virus have been wildly transmitted. The key variants of concern include the Delta strain and the Omicron strain. Although vaccination has helped to reduce SARS‐CoV‐2 spread considerably, the biology of SARS‐CoV‐2 infection remains to be further understood for us to develop effective therapeutics for this disease and possibly future related pandemic. Studies have shown that SARS‐CoV‐2 uses angiotensin‐converting enzyme‐related carboxypeptidase (ACE2) as its major cellular receptor for viral spike protein.^1^ SARS‐CoV‐2 has been detected in almost all human organs, including the lungs, pharynx, heart, liver, brain, kidneys, and digestive system. However, single‐cell sequencing revealed that ACE2 is not expressed or expressed at low levels in multiple human organs such as the lungs and the trachea, and in human tissues including pulmonary and bronchial tissues. How organs and tissues with no or low level of ACE2 expression become infected by SARS‐CoV‐2 remains largely unknown.

Apolipoprotein E (ApoE) is an apolipoprotein that plays an important role in regulating the metabolism and transport of lipids including cholesterol. ApoE is expressed in most organs, including the liver, brain, spleen, lungs, adrenal glands, ovary, kidneys, and muscle, and is circulated in the blood. The mature ApoE protein is a 34‐kDa protein with 299 amino acids containing a 22‐kDa N‐terminal receptor‐binding domain (residues 1–191) and a 10‐kDa C‐terminal lipid‐binding domain (residues 222–299) as well as a hinge region that links the N‐ and C‐terminal domains. As an essential component of lipoprotein particles, ApoE can bind to a variety of cellular receptors, including heparan sulfate proteoglycans, low density lipoprotein receptor (LDLR), very‐low‐density lipoprotein receptor, scavenger receptor class B type I (SR‐BI), and LDLR‐related proteins.^2^ ApoE is known to bind to LDLR on the cell surface to mediate cholesterol and lipid transport.^3^

Previous studies have linked ApoE to COVID and suggested that ApoE may be involved in SARS‐CoV‐2 cellular entry.^4^, ^5^, ^6^ However, how ApoE mediates SARS‐CoV‐2 cellular entry remains unclear. Lipidomic analysis revealed a lipid composition of SARS‐CoV‐2 virions, including cholesterol.^7^ The formation of cholesterol‐rich lipid domains on the viral membrane^7^, ^8^ and the ability of ApoE to bind to cholesterol suggest the ApoE can be part of the SARS‐CoV‐2 lipoviral particles by binding to lipids on the viral membrane. However, there is no direct evidence showing that ApoE is part of the SARS‐CoV‐2 lipoviral particles.

In contrast to the no or low expression of ACE2 in multiple human organs and tissues,^9^, ^10^ ApoE is expressed in most human organs to mediate the transport and cellular uptake of lipids and cholesterol. Considering a cholesterol‐rich lipid composition of SARS‐CoV‐2 virions,^7^, ^8^ we hypothesize that ApoE can be hijacked by SARS‐CoV‐2 to transport viral particles to various tissues and organs, including those that have no or low ACE2 expression, and to facilitate cellular uptake of SARS‐CoV‐2 virions. In this study, we show that ApoE is part of SARS‐CoV‐2 viral particles and can mediate SARS‐CoV‐2 cellular entry by binding to ApoE cellular receptors such as LDLR. An ApoE neutralizing antibody can inhibit SARS‐CoV‐2 infection by blocking SARS‐CoV‐2 cellular entry. The inhibitory effect mediated by the ApoE neutralizing antibody can be observed on both the parental SARS‐CoV‐2 and its variants of concern.

## 2 RESULTS

### 2.1 Preincubation of SARS‐CoV‐2 with an ApoE neutralizing antibody blocks SARS‐CoV‐2 infection

**FIGURE 1:**
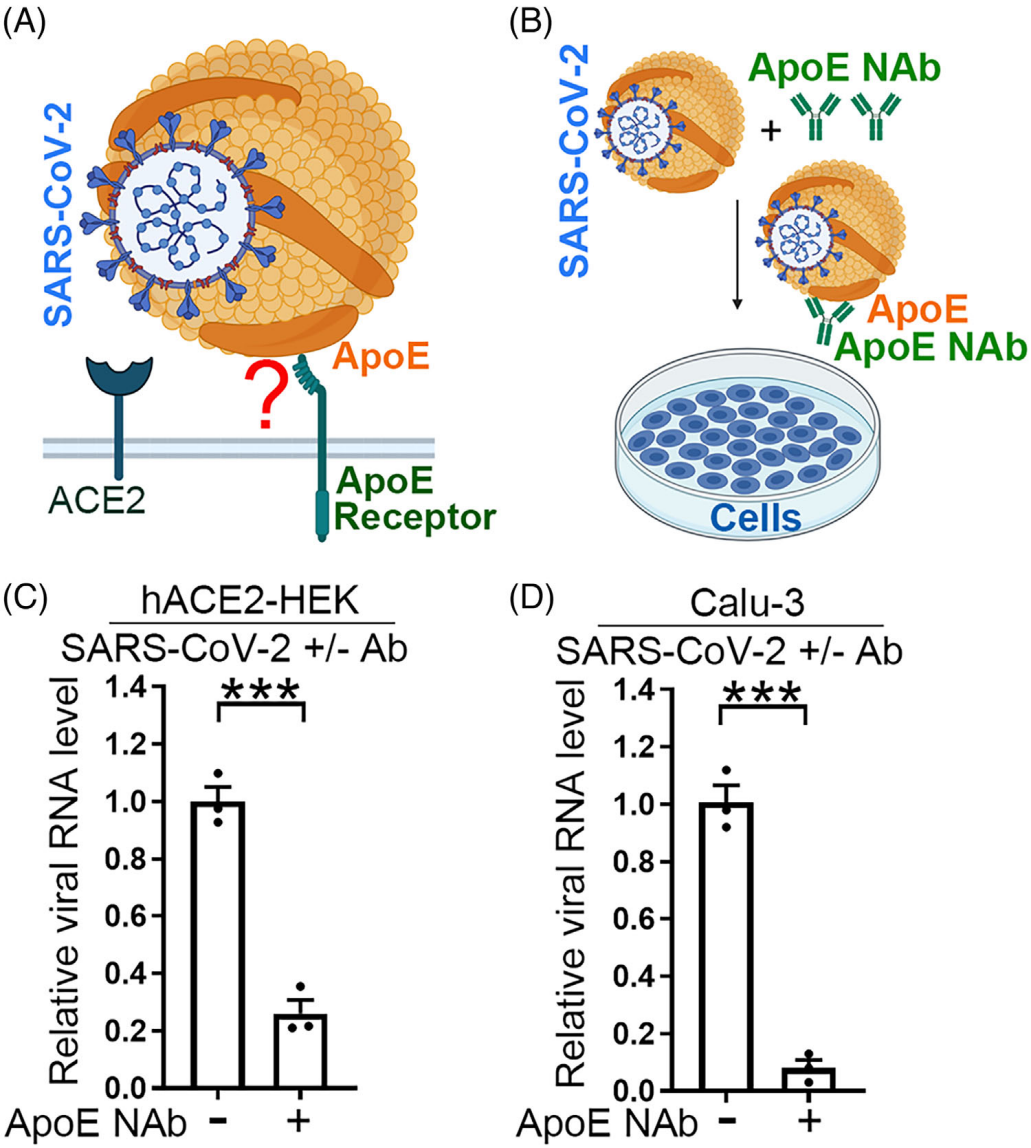
An ApoE neutralizing antibody blocks SARS‐CoV‐2 infection. (A) A schematic illustrating our hypothesis that ApoE on the envelop of lipoviral particles of SARS‐CoV‐2 could facilitate SARS‐CoV‐2 cellular entry by binding to the ApoE receptor on the surface of host cells. (B) A schematic showing our experimental design, including preincubation of SARS‐CoV‐2 with an ApoE neutralizing antibody followed by inoculating the pretreated viruses onto cells. (C and D) Preincubation of SARS‐CoV‐2 virus with an ApoE neutralizing antibody blocks viral infection in hACE2‐HEK cells (C) and Calu‐3 cells (D). The iral N1 RNA level in infected cells was quantified by RT‐PCR. *n* = 3 experimental replicates. Error bars are SE of the mean. ***p < 0.001 by Student's *t*‐test.

To test our hypothesis that ApoE can be part of the lipoviral particles to mediate SARS‐CoV‐2 cellular entry (Figure 1A), we first performed immuno‐electron microscopy analysis of SARS‐CoV‐2 viral particles. The association of ApoE with SARS‐CoV‐2 viral particles was detected by an ApoE‐specific antibody (Figure S1A, right panel). The association of spike with SARS‐CoV‐2 was detected by a spike‐specific antibody and served as the positive control (Figure S1A, left panel). In addition, immunoprecipitation of SARS‐CoV‐2 viral particles with an ApoE‐specific antibody followed by RT‐PCR analysis of viral N1 gene expression revealed enrichment of viral RNA level by the ApoE antibody (Figure S1B, right panel), indicating that the ApoE antibody can pull down SARS‐CoV‐2 virus. Immunoprecipitation of SARS‐CoV‐2 viral particles with the Spike‐specific antibody served as the positive control (Figure S1B, left panel). Next, we preincubated authentic SARS‐CoV‐2 viral particles with an ApoE neutralizing antibody or a control IgG, and then challenged human ACE2‐expressing HEK293T (hACE2‐HEK) or Calu‐3 cells (human lung epithelial cells that can be infected by SARS‐CoV‐2 without overexpression of hACE2) with the pretreated SARS‐CoV‐2 virus (Figure 1B). ApoE neutralizing antibody pretreatment reduced the viral amount substantially, compared with control IgG pretreatment (Figures 1C and D). These results together indicate that ApoE is part of the SARS‐CoV‐2 virions and that the ApoE neutralizing antibody can block SARS‐CoV‐2 infection.

### 2.2 Treatment with an ApoE neutralizing antibody protects cells from SARS‐CoV‐2 infection

**FIGURE 2:**
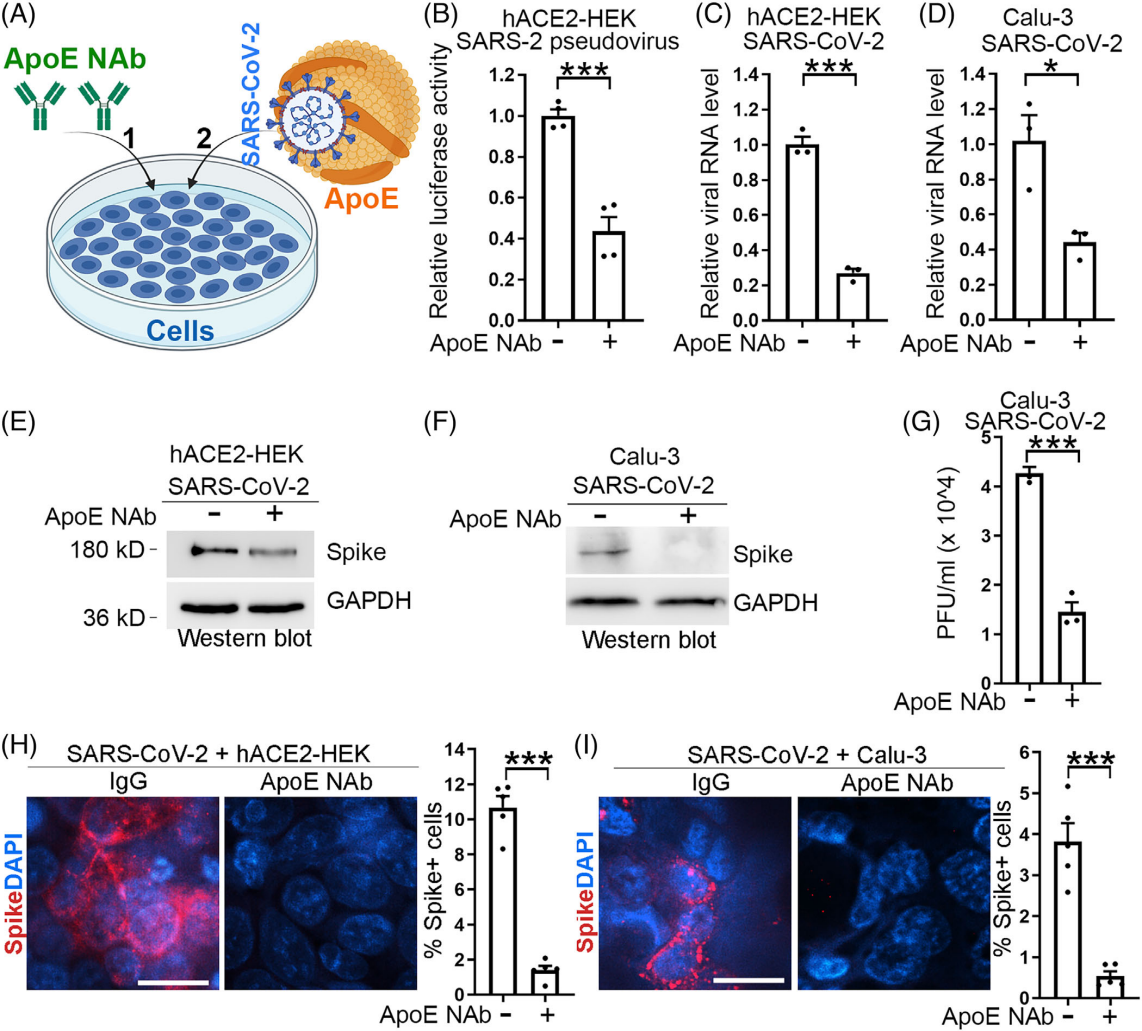
Pretreatment with an ApoE neutralizing antibody protects cells from SARS‐CoV‐2 infection. (A) A schematic showing the experimental design, including pretreatment of cells with an ApoE neutralizing antibody (1) followed by SARS‐CoV‐2 challenge (2). (B) Treatment of hACE2‐HEK cells with an ApoE neutralizing antibody inhibits cellular entry by pseudotyped SARS‐CoV‐2 as evaluated by luciferase activity. *n* = 4 experimental replicates. (C and D) Treatment of hACE2‐HEK cells (C) and Calu‐3 cells (D) with an ApoE neutralizing antibody suppresses SARS‐CoV‐2 infection. The viral amount in infected cells was quantified by RT‐PCR. *n* = 3 experimental replicates. (E and F) Treatment of hACE2‐HEK cells (E) and Calu‐3 cells (F) with an ApoE neutralizing antibody inhibits SARS‐CoV‐2 infection. The viral amount in infected cells was shown by Western blot analysis of spike protein. (G) Treatment of Calu‐3 cells with an ApoE neutralizing antibody inhibits SARS‐CoV‐2 infection. The viral amount in the supernatant of infected cells was quantified by plaque assay. *n* = 3 experimental replicates. (H and I) Treatment of HEK‐ACE2 cells (H) and Calu‐3 cells (I) with an ApoE neutralizing antibody led to reduced percentage of SARS‐CoV‐2‐infected spike+ cells as revealed by immunostaining for spike. Scale bar: 50 μm. *n* = 5 image fields per group for quantification. Error bars are SE of the mean. *p < 0.05 and ***p < 0.001 by Student's *t*‐test for panels B, C, D, G, H, and I.

Having demonstrated a critical role for ApoE in mediating SARS‐CoV‐2 infection, next we asked whether treatment with an ApoE neutralizing antibody could protect cells from SARS‐CoV‐2 infection. To answer this question, we pretreated cells with an ApoE neutralizing antibody followed by SARS‐CoV‐2 challenge in the presence of the ApoE neutralizing antibody (Figure 2A). Pretreatment with the ApoE neutralizing antibody reduced pseudotyped viral amount in hACE2‐HEK cells, as revealed by decreased luciferase activity, compared with pretreatment with control IgG (Figure 2B). ApoE neutralizing antibody pretreatment also reduced the viral amount of authentic SARS‐CoV‐2 in hACE2‐HEK or Calu‐3 cells substantially as revealed by RT‐PCR, compared with control IgG pretreatment (Figures 2C and D). Western blot and immunostaining for spike confirmed the inhibitory effect of the ApoE neutralizing antibody on SARS‐CoV‐2 infection (Figures 2E, F and H, I). The viral amount in the supernatant of infected cells was reduced by pretreatment of cells with the ApoE neutralizing antibody as revealed by the plaque assay (Figure 2G).

**FIGURE 3:**
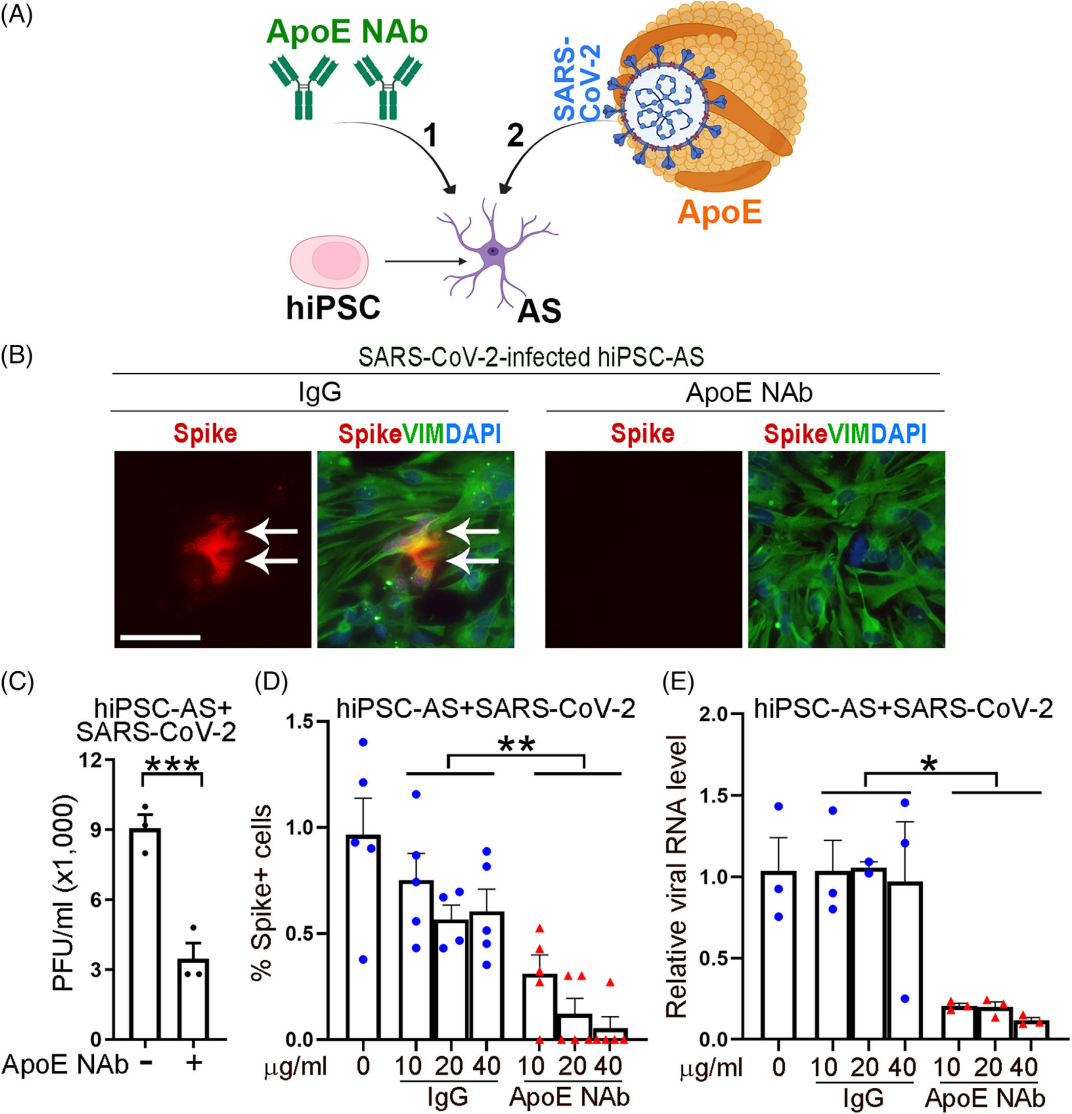
Treatment with an ApoE neutralizing antibody inhibits SARS‐CoV‐2 infection of astrocytes. (A) A schematic showing the experimental design, including pretreatment of human iPSC (hiPSC)‐derived astrocytes (AS) with an ApoE neutralizing antibody (1) followed by SARS‐CoV‐2 challenge (2). (B) Spike and vimentin (VIM) staining of hiPSC‐derived AS treated with a control IgG or an ApoE neutralizing antibody followed by SARS‐CoV‐2 infection. Scale bar: 50 μm. Arrows indicate Spike+ cells. (C) Treatment of hiPSC‐AS with an ApoE neutralizing antibody inhibits SARS‐CoV‐2 infection. The viral amount in the supernatant of infected cells was quantified by plaque assay. *n* = 3 experimental replicates. (D) Quantification of Spike+ cells in IgG control or ApoE neutralizing antibody treated cells. *n* = 5 image fields per group. (E) RT‐PCR analysis of viral RNA levels in IgG control or ApoE neutralizing antibody treated cells. The level of viral RNA was compared and normalized to the level in control condition (0 μg/mL). *n* = 3 experimental replicates. Error bars are SE of the mean. ***p < 0.001 by Student's *t*‐test for panels C. *p < 0.05 and **p < 0.01 by one‐way ANOVA test for panels D and E.

In addition to hACE2‐HEK or Calu‐3 cells, we further tested the protective effect of the ApoE neutralizing antibody on human iPSC‐derived astrocytes subjected to SARS‐CoV‐2 infection. Pretreatment of human iPSC‐derived astrocytes with the ApoE neutralizing antibody followed by SARS‐CoV‐2 exposure also suppressed SARS‐CoV‐2 infection (Figure 3A), as revealed by the decreased percentage of viral spike‐positive cells (Figures 3B and D), reduced viral amount (Figure 3C), and lowered viral RNA levels (Figure 3E), compared with control IgG pretreatment. Taken together, these data provide strong evidence that an ApoE neutralizing antibody can protect human cells from SARS‐CoV‐2 infection, therefore can serve as a promising antiviral therapy for COVID.

### 2.3 The ApoE neutralizing antibody blocks infection by the SARS‐CoV‐2 Delta strain

**FIGURE 4:**
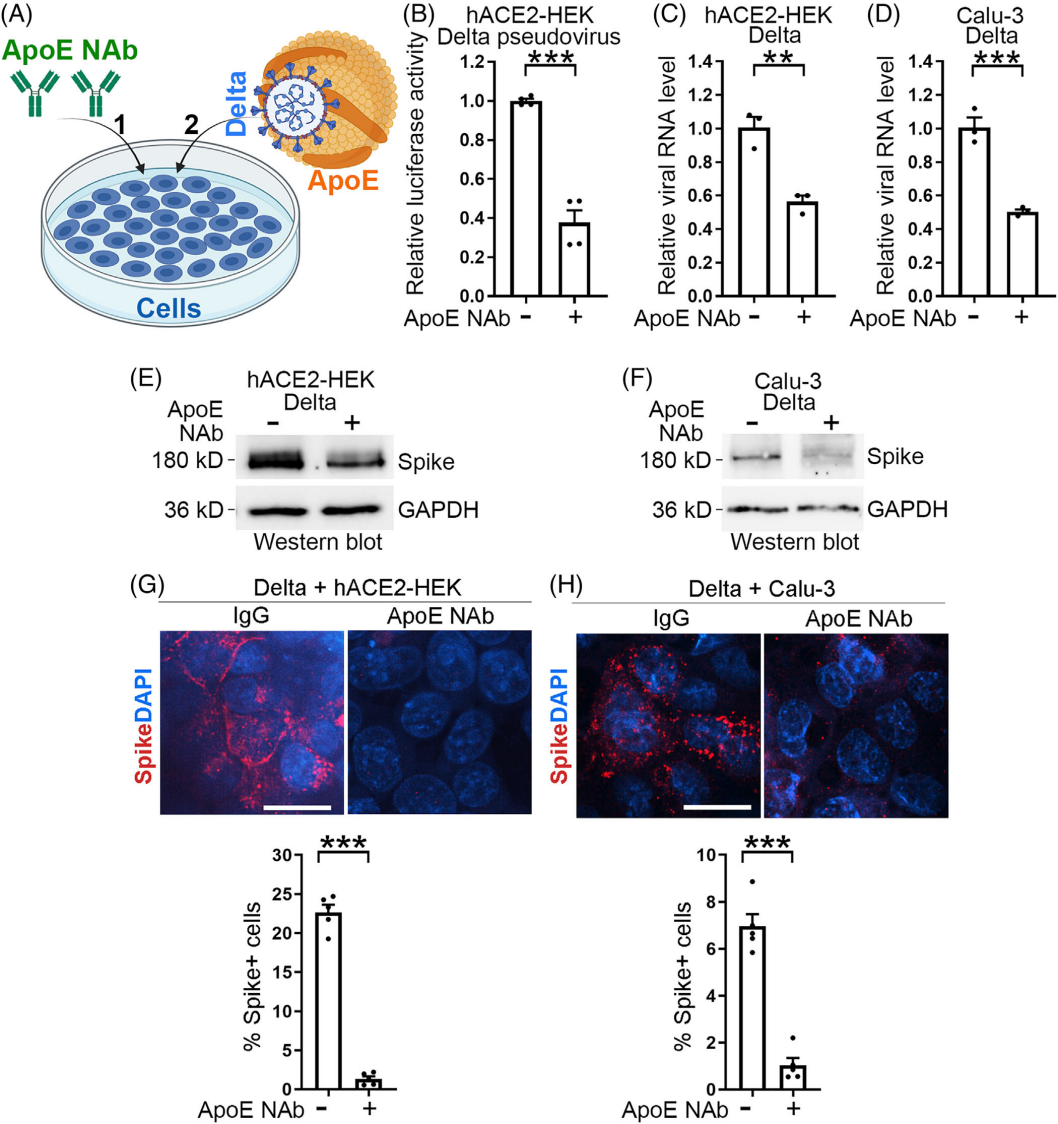
An ApoE neutralizing antibody blocks infection by SARS‐CoV‐2 Delta strain. (A) A schematic showing the experimental design, including cell pretreatment by ApoE neutralizing antibody (1) followed by SARS‐CoV‐2 Delta strain challenge (2). (B) Treatment of hACE2‐HEK cells with an ApoE neutralizing antibody inhibits cellular entry by pseudotyped Delta SARS‐CoV‐2, as evaluated by luciferase activity. *n* = 4 experimental replicates. (C and D) Treatment of hACE2‐HEK cells (C) and Calu‐3 cells (D) with an ApoE neutralizing antibody suppresses SARS‐CoV‐2 Delta strain infection. The viral RNA level in infected cells was quantified by RT‐PCR. *n* = 3 experimental replicates. (E and F) Treatment of hACE2‐HEK cells (E) or Calu‐3 cells (F) with an ApoE neutralizing antibody inhibits SARS‐CoV‐2 Delta strain infection. The viral amount in infected cells was shown by Western blot analysis of spike protein. (G, H) Treatment of hACE2‐HEK cells (G) or Calu‐3 cells (H) with an ApoE neutralizing antibody led to reduced percentage of SARS‐CoV‐2‐infected spike+ cells as revealed by immunostaining for spike. Scale bar: 50 μm. *n* = 5 image fields per group for quantification. Error bars are SE of the mean. **p < 0.01 and ***p < 0.001 by Student's *t*‐test for panels B, C, D, G, and H.

As a potent SARS‐CoV‐2 variant of concern emerged after the parental strain, the Delta strain of SARS‐CoV‐2 is highly contagious. We next tested if the ApoE neutralizing antibody could block the infection by the SARS‐CoV‐2 Delta strain (Figure 4A). hACE2‐HEK cells were pretreated with an ApoE neutralizing antibody or a control IgG, and then subjected to pseudotyped or authentic SARS‐CoV‐2 Delta strain challenge. ApoE neutralizing antibody pretreatment reduced both pseudotyped (Figure 4B) and authentic SARS‐CoV‐2 Delta viral amount substantially (Figures 4C–H), compared with control IgG pretreatment. Specifically, pretreatment of hACE2‐HEK cells with the ApoE neutralizing antibody protected cells from infection by the SARS‐CoV‐2 Delta virus as revealed by the substantially reduced viral RNA level in cells pretreated with the ApoE neutralizing antibody, compared with that in cells pretreated with a control IgG (Figure 4C). A similar protective effect by the ApoE neutralizing antibody was detected in Calu‐3 cells (Figure 4D). In addition to decreasing SARS‐CoV‐2 Delta viral RNA level (Figures 4C and D), pretreatment with the ApoE neutralizing antibody also reduced the spike protein level of SARS‐CoV‐2 Delta virus, compared with pretreatment with a control IgG (Figures 4E and F). Immunostaining for the viral spike protein further confirmed that pretreatment with the ApoE neutralizing antibody could protect cells from Delta virus infection, as revealed by the reduced percentage of cells infected by the SARS‐CoV‐2 Delta strain in both hACE2‐HEK cells (Figure 4G) and Calu‐3 cells (Figure 4H) pretreated with the ApoE neutralizing antibody, compared with that in cells pretreated with a control IgG. These results indicate that the ApoE neutralizing antibody can protect cells from infection by the SARS‐CoV‐2 Delta strain.

### 2.4 The ApoE neutralizing antibody blocks infection by the SARS‐CoV‐2 Omicron strain

**FIGURE 5:**
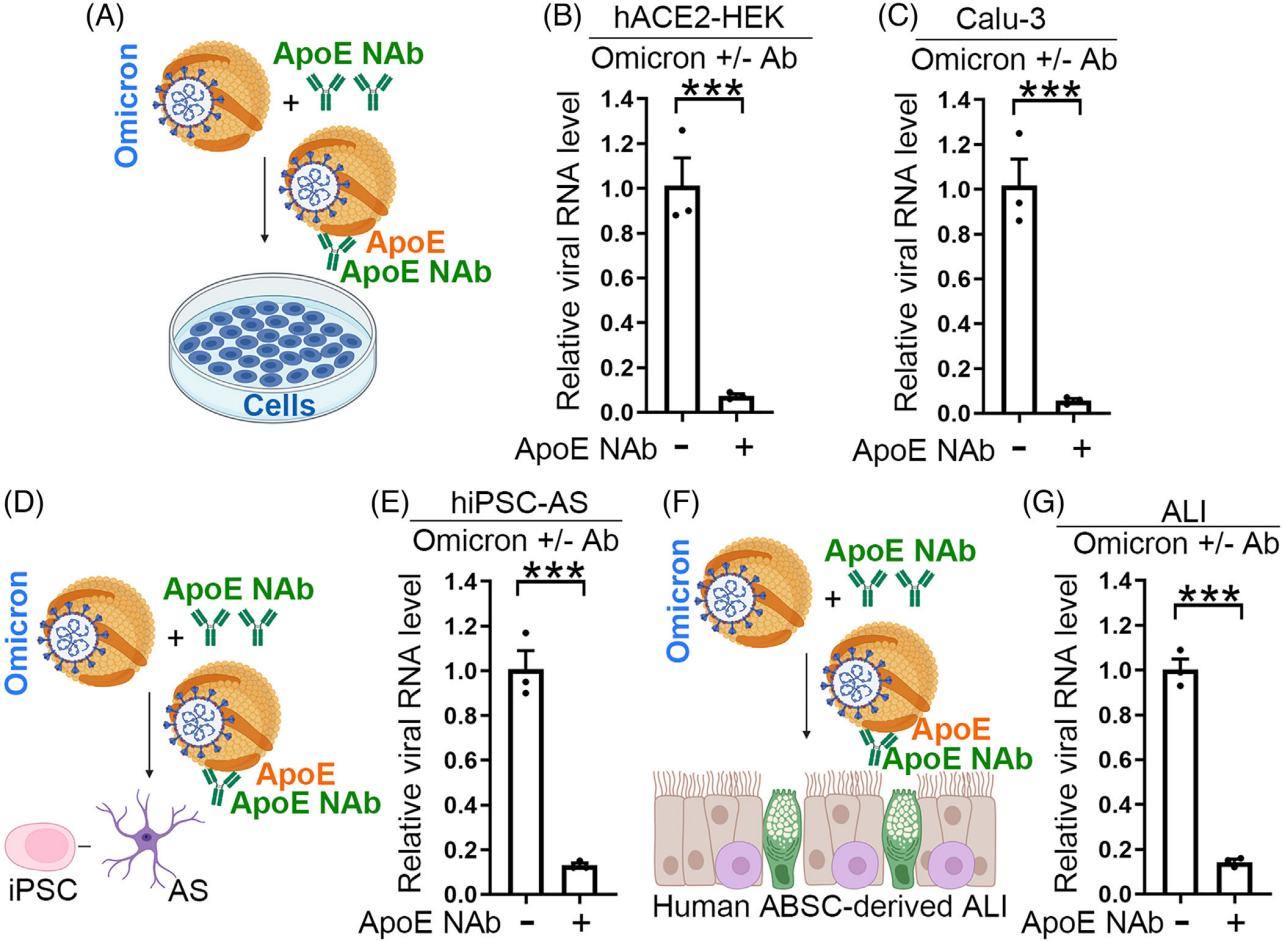
An ApoE neutralizing antibody blocks infection by the SARS‐CoV‐2 Omicron strain. (A) A schematic showing the experimental design, including preincubation of SARS‐CoV‐2 Omicron virus with an ApoE neutralizing antibody followed by inoculation of the pretreated viruses onto cells. (B and C) Preincubation of SARS‐CoV‐2 Omicron virus with an ApoE neutralizing antibody blocks viral infection in hACE2‐HEK cells (B), and Calu‐3 cells (C). The viral N1 RNA level in infected cells was quantified by RT‐PCR. *n* = 3 experimental replicates. Error bars are SE of the mean. ***p < 0.001 by Student's *t*‐test. (D) A schematic showing the experimental design, including preincubation of SARS‐CoV‐2 Omicron virus with an ApoE neutralizing antibody followed by inoculation of the pretreated viruses onto iPSC derived astrocytes (AS). (E) Preincubation of SARS‐CoV‐2 Omicron virus with an ApoE neutralizing antibody blocks viral infection in iPSC derived astrocytes (AS). The viral N1 RNA level in infected cells was quantified by RT‐PCR. *n* = 3 experimental replicates. Error bars are SE of the mean. ***p < 0.001 by Student's *t*‐test. (F) A schematic showing the experimental design, including preincubation of SARS‐CoV‐2 Omicron with an ApoE neutralizing antibody followed by inoculation of the pretreated viruses onto human airway basal stem cells (ABSC)‐derived air–liquid interface (ALI) cultures. (G) Viral N1 RNA level in infected cells from (F) was quantified by RT‐PCR. *n* = 3 experimental replicates. Error bars are SE of the mean. ***p < 0.001 by Student's *t*‐test.

The Omicron strain of SARS‐CoV‐2 became a predominant variant in circulation after Delta and posed an eminent threat to public health. We then tested if the ApoE neutralizing antibody can block infection by the SARS‐CoV‐2 Omicron strain. The Omicron virus was pretreated with an ApoE neutralizing antibody or a control IgG, then a panel of cells including hACE2‐HEK, Calu‐3, and human iPSC‐derived astrocytes were infected with the pretreated Omicron virus (Figure 5A). The ApoE neutralizing antibody blocked the infection of hACE2‐HEK, Calu‐3, and astrocytes by Omicron virus substantially (Figures 5B–E), similar to the infection by the parental SARS‐CoV‐2 and the Delta strain. To test the effect of the ApoE neutralizing antibody on SARS‐CoV‐2 infection in a more disease‐relevant system, we used the human primary airway basal stem cell (ABSC)‐derived air–liquid interface (ALI) organoid model. The ALI organoids were challenged by the SARS‐CoV‐2 Omicron virus pretreated with the ApoE neutralizing antibody or the IgG control (Figure 5F). The ApoE neutralizing antibody blocked the infection of the ALI organoid cultures by the SARS‐CoV‐2 Omicron variant efficiently (Figure 5G). These data together demonstrate that the ApoE neutralizing antibody can block infection by the SARS‐CoV‐2 Omicron strain effectively.

### 2.5 ApoE binds to its cellular receptor LDLR to facilitate SARS‐CoV‐2 infection

**FIGURE 6:**
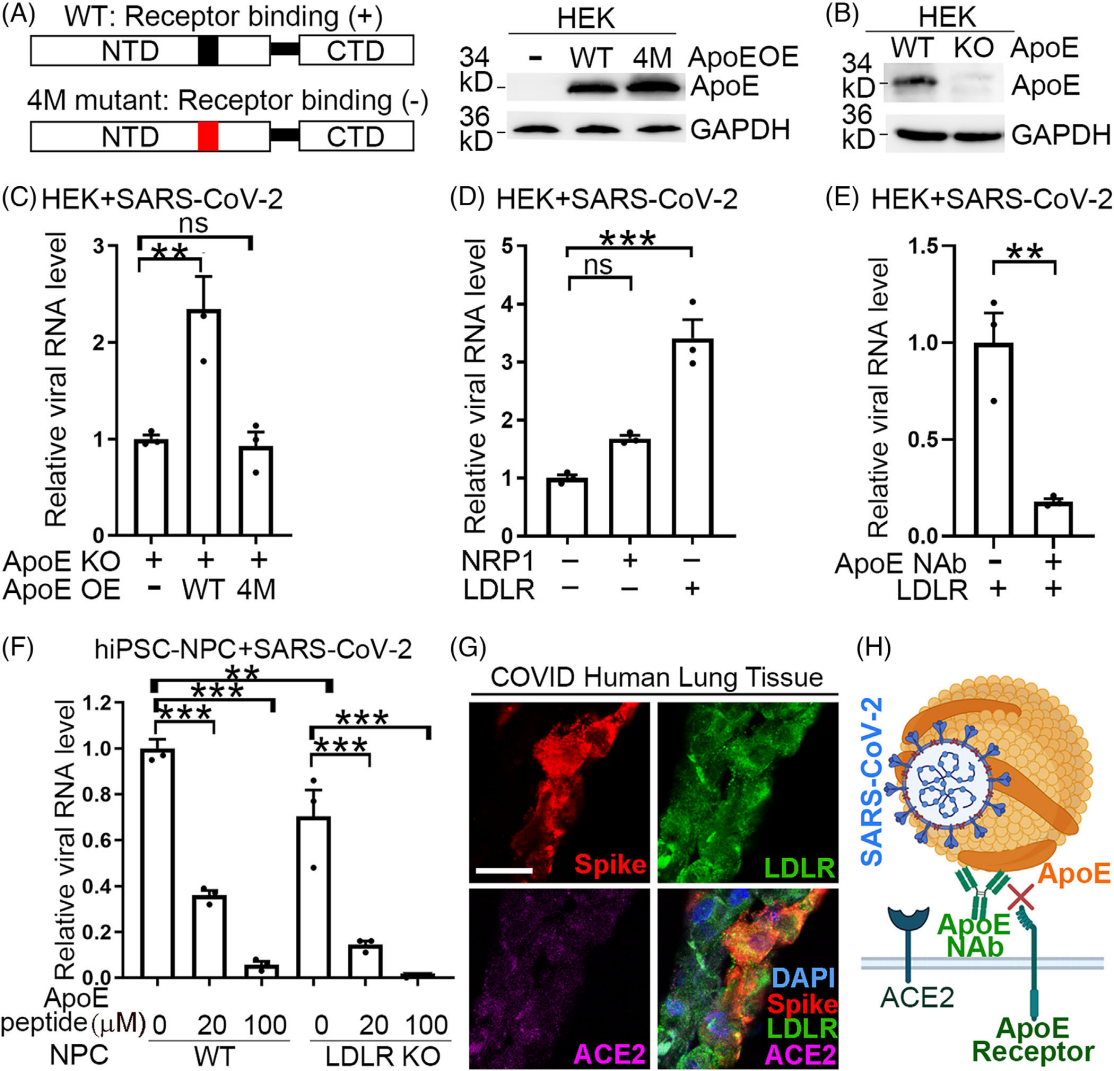
LDLR facilitates SARS‐CoV‐2 infection. (A) A schematic of the WT or the 4 M mutant ApoE with mutation in the receptor‐binding region (red) in the N‐terminal domain (NTD) is shown in the left panel. CTD: C‐terminal domain. Western blot of ApoE in HEK cells with or without overexpression of the WT or the 4 M mutant ApoE is shown in the right panel. (B) Western blot of ApoE in HEK cells with or without ApoE knockout (KO). (C) Overexpression of the WT but not the 4 M mutant ApoE in ApoE KO HEK cells increases SARS‐CoV‐2 infection as revealed by RT‐PCR. *n* = 3 experimental replicates. **p < 0.01, ns: not statistically significant (*p* > 0.05) by one‐way ANOVA. Error bars are SE of the mean. (D) Overexpression of LDLR on HEK cells facilitates SARS‐CoV‐2 infection. Overexpression of NRP1 in HEK cells was included as a control. The viral RNA level in SARS‐CoV‐2‐infected cells was quantified by RT‐PCR. *n* = 3 experimental replicates. Error bars are SE of the mean. ***p < 0.001. ns: not statistically significant (*p* > 0.05) by one‐way ANOVA. (E) Treatment of LDLR‐HEK cells with an ApoE neutralizing antibody inhibits SARS‐CoV‐2 infection. The viral RNA level in infected cells was quantified by RT‐PCR. *n* = 3 experimental replicates. Error bars are SE of the mean. **p < 0.01 by Student's *t*‐test. (F) Treatment of wild type (WT) hiPSC‐derived neural progenitor cells (NPC) or LDLR KO hiPSC‐derived NPC with an ApoE mimetic peptide inhibits SARS‐CoV‐2 infection. Viral RNA level in infected cells was quantified by RT‐PCR. *n* = 3 experimental replicates. Error bars are SE of the mean. **p < 0.01 and ***p < 0.001 by one‐way ANOVA followed by Dunnett's multiple comparisons test. (G) Immunohistochemical staining for Spike, LDLR, and ACE2 on human lung tissues from COVID patient. Scale bar: 20 μm. (H) A schematic showing inhibition of SARS‐CoV‐2 infection by an ApoE neutralizing antibody via blocking the interaction of ApoE on the SARS‐CoV‐2 lipoviral particles with an ApoE receptor on host cells.

To determine how ApoE mediates SARS‐CoV‐2 entry into host cells, we overexpressed the wild type (WT) or the 4 M mutant ApoE (with mutation of arginine at residues 145, 147, and 150 and lysine at residue 146 to alanine) that has been shown to lose its receptor binding in the ApoE knockout (KO) cells (Figures 6A and B). ApoE was detected on SARS‐CoV‐2 viral particles that were immunopurified using a spike‐specific antibody from the viral supernatant of SARS‐CoV‐2‐infected ApoE KO HEK cells that overexpressed Flag‐tagged WT or 4 M mutant ApoE (Figure S2A). Overexpression of the WT but not the 4 M mutant ApoE enhanced SARS‐CoV‐2 infection substantially as revealed by increased viral RNA level in infected cells (Figure 6C). These results indicate that the interaction of ApoE with its cellular receptor(s) plays an important role in mediating SARS‐CoV‐2 viral infection.

LDLR is a well‐known receptor for ApoE and has been shown to mediate hepatitis C virus (HCV) infection,^11^ we asked if LDLR can serve as the ApoE cellular receptor to facilitate SARS‐CoV‐2 infection. To answer this question, HEK293T (HEK) cells were transfected with LDLR and then challenged with authentic SARS‐CoV‐2. HEK cells transfected with neuropilin‐1 (NRP1), a reported SARS‐CoV‐2 cofactor, were included as controls. An increase of SARS‐CoV‐2 viral amount was detected in LDLR‐overexpressing HEK cells, compared with mock‐transfected cells, while only a mild increase was detected in NRP1‐overexpressing cells (Figures 6D and S2B, S2C).

Pretreatment of the LDLR‐overexpressing HEK cells with an ApoE neutralizing antibody inhibited SARS‐CoV‐2 infection, compared with control IgG pretreatment (Figure 6E), indicating that LDLR‐mediated SARS‐CoV‐2 infection is ApoE‐dependent. KO of LDLR in hiPSC‐derived neural progenitor cells (NPCs), a cell type that expressed LDLR but not ACE2 (Figure S3A), reduced SARS‐CoV‐2 infection (Figure 6F). Treatment of WT hiPSC‐NPCs with an ApoE mimetic peptide, which blocks the interaction of ApoE with its receptors,^12^ also inhibited SARS‐CoV‐2 infection. Treatment of LDLR KO hiPSC‐NPCs with the ApoE mimetic peptide further reduced SARS‐CoV‐2 infection (Figure 6F). These results together indicate that the interaction between ApoE and ApoE receptors including LDLR plays an important role in mediating SARS‐CoV‐2 infection. Moreover, to study the interaction of ApoE with SARS‐CoV‐2 entry receptor and cofactors, we overexpressed ApoE with LDLR or NRP1 in HEK cells that overexpressed ACE2 and TMPRSS2, and investigated pseudotyped SARS‐CoV‐2 viral entry using a luciferase reporter assay. We found that overexpression of ApoE, LDLR, or NRP1 facilitated viral entry in HEK cells overexpressed with ACE2 and the transmembrane protease, serine 2 (TMPRSS2), and ApoE further enhanced LDLR or NRP1‐facilitated viral entry (Figures S3B and C).

We further examined the expression of LDLR in cells and human tissues. While LDLR expression was detected in human iPSC‐derived astrocytes and human iPSC‐derived NPCs clearly, no expression of ACE2 was detected in these cells (Figures S3A and S4A). This result suggests that LDLR could be a cellular receptor for ApoE to mediate SARS‐CoV‐2 infection in cells that do not express ACE2. The expression of LDLR was also detected in hACE2‐HEK, Calu‐3, and Vero cells. In contrast, the expression of ACE2 was only detected in hACE2‐HEK and weakly in Calu‐3 but barely in other cells (Figure S4A), suggesting that LDLR could mediate SARS‐CoV‐2 infection as a cellular receptor for ApoE, especially in cells that lack ACE2 expression thus cannot use ACE2 as the host receptor for SARS‐CoV‐2 entry.

We next examined the expression of LDLR in human tissues from COVID patients. We detected SARS‐CoV‐2 Spike‐positive cells in COVID but not control human lung tissues (Figures 6G and S4B). The Spike‐positive cells expressed strong membrane LDLR but had no or low ACE2 expression (Figure 6G). This result indicates that SARS‐CoV‐2 can infect LDLR‐positive but ACE2‐negaive cells in human lung tissues. Taken together, data generated from this study support a model in which SARS‐CoV‐2 can infect human cells by binding of ApoE on SARS‐CoV‐2 lipoviral particles to ApoE cellular receptors (such as LDLR) on host cells. Moreover, an ApoE neutralizing antibody can block the infection by SARS‐CoV‐2 and its variants of concern (Figure 6H).

## 3 DISCUSSION

In this study, we demonstrate that ApoE is part of SARS‐CoV‐2 viral particles, and that SARS‐CoV‐2 infection of host cells can be blocked potently by a neutralizing antibody against ApoE. Treatment with an ApoE neutralizing antibody blocks the entry of SARS‐CoV‐2 virus into hACE2‐HEK cells, human iPSC‐derived astrocytes, human lung epithelial Calu‐3 cells, and human primary ALI organoid cultures. Moreover, the ApoE neutralizing antibody is very effective in inhibiting the infection of not only the parental SARS‐CoV‐2 but also its variants including the Delta strain and the Omicron strain. Thus, this study not only highlights the interaction of ApoE on the viral particles to ApoE receptor(s) on host cells in facilitating SARS‐CoV‐2 cellular entry, but also provide a potential therapeutic strategy for combating COVID by inhibiting SARS‐CoV‐2 infection using an ApoE neutralizing antibody. An advantage of targeting ApoE instead of the viral spike protein is that the effectiveness of ApoE antibodies will likely not be affected by mutation of the viral genome in newly emerged SARS‐CoV‐2 variants/subvariants.

As an early step during viral life circle, cellular entry is critical for viral infection, thus can be an important target for blocking viral infection. Our understating of SARS‐CoV‐2 cellular entry is continuously evolving. Besides ACE2 that was identified as the first SARS‐CoV‐2 receptor,^1^, ^13^ additional receptors or cofactors have been identified for SARS‐CoV‐2, including NRP1^14^, ^15^ and AXL,^16^ that can mediate SARS‐CoV‐2 cellular entry. Recent findings that lipids and cholesterol are critical for SARS‐CoV‐2 cellular entry as part of the lipoviral particles^7^, ^8^, ^17^ provide further insights into the entry of SARS‐CoV‐2 into host cells. Cholesterol‐dependent mechanisms are also critical for cellular entry and infection by influenza virus.^18^, ^19^ Because ApoE is an apolipoprotein that binds to lipids and cholesterol to mediate their cellular uptake,^20^, ^21^ we hypothesize that ApoE can be part of the lipoviral particles by binding to lipids and cholesterol on the lipoviral particles to mediate SARS‐CoV‐2 cellular entry. In this study, we found that preincubation of SARS‐CoV‐2 with an antibody against ApoE could block the entry of virus to host cells effectively, supporting our hypothesis that ApoE acts as part of the lipoviral particles to mediate cellular entry of SARS‐CoV‐2. An advantage of using a cellular protein ApoE for entry into host cells by SARS‐CoV‐2 is to evade the host immune system. The host proteins on virions can allow viruses to escape the host immune response, thus helping viruses to establish infections.

The main steps of cellular entry by viruses include attachment of viruses to cell surface receptors and the delivery of the viral genome to the cytoplasm of host cells.^22^ Identification of routes of entry and receptors is important for understanding viral tropism and pathogenicity to develop effective antiviral therapies.^22^ We show here that LDLR, a well‐known cellular receptor for ApoE,^23^, ^24^ can be a facilitating factor and a cellular receptor for SARS‐CoV‐2 infection. Overexpression of LDLR in HEK293T cells increased SARS‐CoV‐2 infection substantially, in a manner that is more potent than overexpression of NRP1, a previously identified receptor for SARS‐CoV‐2.^14^, ^15^ Treatment of LDLR‐overexpressing HEK cells with an ApoE neutralizing antibody blocked SARS‐CoV‐2 infection largely, suggesting that binding of ApoE to LDLR is important for SARS‐CoV‐2 infection. Mutation of the ApoE receptor binding domain reduces SARS‐CoV‐2 infection, further supporting the importance of ApoE–receptor interaction in SARS‐CoV‐2 infection. ApoE has also been shown to mediate HCV binding to target cells as a structural component of the viral particles^25^ and facilitate HCV cellular entry through binding to its cellular receptor LDLR.^11^ Our previous study demonstrated that the ApoE isoforms are associated with SARS‐CoV‐2 neurotropism.^4^ However, how ApoE regulates SARS‐CoV‐2 infection remained unknown. In this study, we uncovered an ApoE‐dependent route for SARS‐CoV‐2 entry through binding of ApoE on lipoviral particles to its cellular receptors on host cells. While we have demonstrated that LDLR can facilitate SARS‐CoV‐2 infection, we do not rule out the possibility that ApoE may also interact with other cellular receptors to mediate SARS‐CoV‐2 cellular entry. For example, the scavenger receptor, class B type 1 (SR‐B1) has been shown to facilitate SARS‐CoV‐2 entry by binding to high‐density lipoproteins (HDL).^26^ However, the component in HDL that mediates the SR‐B1 effect remains to be identified. Our study suggests that ApoE in HDL can facilitate SARS‐CoV‐2 entry by binding to cellular receptors such as SR‐B1, a hypothesis that can be tested in the future. Variants of ApoE isoforms have been shown to differentially impact SARS‐CoV‐2 cellular infection and COVID‐19 outcome in SARS‐CoV‐2 patients and human iPSC‐based models as well as a mouse model.^4^, ^5^, ^6^ We mutated the ApoE receptor‐binding domain in the ApoE3 isoform in this study. It will be interesting to compare the effects in the context of different ApoE isoform variants and test isoform‐specific ApoE neutralizing antibodies in blocking SARS‐CoV‐2 infection in the future.

Uncovering mechanisms that underlie the broad tissue tropism of SARS‐CoV‐2 is critical for developing effective therapeutics for COVID. ACE2 has been identified as the host receptor for the spike protein of SARS‐CoV‐2 to mediate SARS‐CoV‐2 cellular entry.^1^, ^13^ However, while SARS‐CoV‐2 can be detected in almost all human organs, ACE2 could not been detected in all organs.^9^, ^10^ We show here that ApoE can mediate SARS‐CoV‐2 entry into host cells through binding to its cellular receptor. ApoE is expressed in most organs, including the liver, brain, spleen, lung, adrenal glands, ovary, kidney, and muscle, and is circulated in the blood.^27^, ^28^, ^29^, ^30^, ^31^ ApoE can bind to various receptors including LDLR to mediate lipid and cholesterol uptake and transport to a variety of cells and tissues.^32^ Such a mechanism can be used to spread SARS‐CoV‐2 to many human organs, including those that do not express ACE2 and other receptors that have been identified for SARS‐CoV‐2, including NRP1^14^, ^15^ and AXL.^16^ Blood has served as one of the main routes for viral transmission. The presence of ApoE on lipoviral particles can facilitate the circulation of viral particles in the blood and spread to tissues and organs all over the body through the ApoE–receptor interactions, mimicking ApoE‐mediated transport of lipids.^20^, ^33^

The presence of ApoE on SARS‐CoV‐2 viral particles provides an opportunity to block viral infection using ApoE neutralizing antibodies. Because ApoE is less prone to mutations than the viral spike‐encoding gene, the strategy of targeting ApoE using its neutralizing antibodies can be less affected by the emergence of new viral variants resulted from the mutation of the viral spike gene. In this study, we demonstrated that an ApoE neutralizing antibody could block the infection of SARS‐CoV‐2 and its Delta and Omicron variants effectively. Thus, ApoE neutralizing antibodies can provide broad protection from infection by continuously emerging SARS‐CoV‐2 variants of concern, overcoming the limitation of antibodies targeting the spike protein for which the encoding gene can be frequently mutated. It is possible that ApoE may play important roles not only in SARS‐CoV‐2 cellular entry, but also in other steps of the viral life cycle including virion assembly and egression, and in cellular response to viral infection, which can be important aspects for future studies to further develop the ApoE‐targeted strategy for inhibition of SARS‐CoV‐2 infection and consequent cellular damage.

While this study has demonstrated robust efficacy of the ApoE neutralizing antibody in blocking SARS‐CoV‐2 cellular entry in vitro, a limitation of the study is that there is no data on in vivo efficacy. Moreover, a careful safety study needs to be performed before we can bring this strategy to the clinic.

In summary, this study provides evidence that ApoE is part of the SARS‐CoV‐2 lipoviral particles and uncovers a new route of cellular entry by SARS‐CoV‐2 via ApoE–receptor interactions, which can be used to explain why SARS‐CoV‐2 has been detected in almost all human organs, although ACE2 is not expressed or expressed at low levels in multiple human organs and tissues, thus providing a plausible explanation for the broad tissue tropism of SARS‐CoV‐2. Moreover, in this study, we have provided robust evidence that an ApoE neutralizing antibody can inhibit SARS‐CoV‐2 infection. An ApoE neutralizing antibody can inhibit SARS‐CoV‐2 entry by blocking the interaction of ApoE on lipoviral particles with ApoE receptors on host cells, or preventing the spike‐ACE2 interactions through allosteric hindrance resulted from binding of the ApoE antibody to ApoE on the lipoviral particles. Because ApoE neutralizing antibodies can inhibit SARS‐CoV‐2 infection by both parental virus and its variants of concern, these antibodies have the potential to be developed into effective therapies for broad protection from COVID despite the evolvement of new variants.

## 4 MATERIALS AND METHODS

### 4.1 Cell culture

HEK293T cells (HEK) and Vero cells were cultured in DMEM medium and Calu‐3 cells were cultured in Minimum Essential Medium as previously described.^34^ AG14048 (Catalog # AG14048) and AG06809 (Catalog # AG06869) fibroblasts were obtained from Coriell and reprogrammed to iPSCs as we described previously.^4^, ^35^, ^36^, ^37^, ^38^ The LDLR KO iPSCs were generated by electroporation of Cas9‐sgRNA ribonucleoproteins into AG06809 iPSCs followed by KO clone screening via sanger sequencing. The sgRNA sequence for LDLR is 5′ GAA TTC GTC AGG GCG ACA GG 3′. The WT or KO iPSCs were used to differentiate into NPCs or astrocytes following the protocols we described previously.^4^, ^38^ ALI cultures derived from primary human proximal ABSCs were used as described previously.^39^ Briefly, 24‐well 6.5 mm trans‐wells with 0.4 mm pore polyester membrane inserts were used for culturing ALI cells. 500 μL ALI media (PneumaCult™‐ALI Medium; STEMCELL Technologies) was used in the basal chamber for ALI cultures and cells were cultured at 37°C with 5% CO_2_. No mycoplasma contamination in cultures was confirmed by MycoAlert PLUS Mycoplasma Detection Kit (Lonza; Catalog # LT07‐318).

### 4.2 Plasmids

The hACE2‐encoding plasmid (Addgene #1786) was used to clone hACE2‐expressing lentiviral vector. The NRP1‐expressing (Addgene #158384), LDLR‐expressing (Addgene #162717), TMPRSS2‐expressing (Addgene #154963), and ApoE3‐expressing (Addgene #87086) plasmids were used to overexpress relevant proteins by transfection into target cells through calcium phosphate precipitation. The ApoE3‐expressing plasmid (Addgene #87086) was used to generate mutant ApoE 4 M with mutations of arginine to alanine at residues 145, 147, and 150 and lysine to alanine at residue 146.

### 4.3 Viral preparation

The SARS‐CoV‐2 pseudovirus was prepared as previously described.^34^ Briefly, the SARS‐CoV‐2 spike pseudovirus and the SARS‐CoV‐2 Delta strain spike pseudovirus were prepared by transfecting the plasmid pcDNA3.1‐SARS2‐Spike (Addgene #145032) or plasmid pcDNA3.3‐SARS‐B.1.617.2 Spike (Addgene #172320), respectively, together with plasmids pMDL, pREV, and pHIV7‐eGFP‐ffLuc into HEK293T cells. The authentic SARS‐CoV‐2 (Isolate USA‐WA1/2020) and SARS‐CoV‐2 Delta strain (Isolate hCoV‐19/USA/MD‐HP05647/2021, Lineage B.1.617.2) was prepared as previous described.^4^, ^40^, ^41^ The SARS‐CoV‐2 Omicron variant (NR‐56475, Isolate hCoV‐19/USA/HI‐CDC‐4359259‐001/2021) used in this study was obtained through BEI Resources. The following reagent was deposited by the Centers for Disease Control and Prevention and obtained through BEI Resources, NIAID, NIH: SARS‐Related Coronavirus 2, Isolate USA‐WA1/2020, NR‐52281.

### 4.4 Inhibition of SARS‐CoV‐2 infection by neutralizing antibody

For testing antibody incubation in blocking viral infection, the authentic SARS‐CoV‐2 (100 pfu) was incubated with 20 μg/mL ApoE neutralizing antibody (Millipore Sigma; Catalog # 178479) or 20 μg/mL IgG (R&D Systems; Catalog # AB‐108‐C) for 1 hour (h) at room temperature. Antibody incubated virus was then added to relevant cells for infection of 24 h. Virus containing medium was removed and cells were harvested for analysis. For infection of ALI cultures by antibody‐incubated Omicron virus, the virus‐antibody mixture was inoculated on to the apical side of the ALI cells and the cells were incubated for 1 h to allow the virus to adsorb onto the cells. Subsequently, the viral inoculum was removed from the apical side and the cells were incubated for an additional 24 h for RNA analysis.

For testing antibody treatment in protecting cells from viral infection, relevant cells were pretreated with 20 μg/mL ApoE neutralizing antibody (Millipore Sigma; Catalog # 178479) or 20 μg/mL IgG (R&D Systems; Catalog # AB‐108‐C), or 20 μM or 100 μM ApoE mimetic peptide (COG 133; ApexBio Technology; Catalog # A1131) for 2 h. For astrocyte treatment, a dose range from 0 to 40 μg/mL ApoE neutralizing antibody or IgG was included. The authentic SARS‐CoV‐2 or SARS‐CoV‐2 Delta strain was then added to cells (at MOI 0.1 for HEK cells and Calu‐3 cells, at MOI 1 for astrocytes and NPCs) for infection of 24 h for HEK, 48 h for Calu‐3 cells and NPCs, and of 3 days for astrocytes. Virus‐containing medium was harvested for plaque assay to determine viral amount by infecting hACE2‐Vero cells, and cells were harvested for RNA and protein analyses.

### 4.5 RT‐PCR

RT‐PCR analysis was performed as previously described.^34^ Briefly, SARS‐CoV‐2 detection primer: nCoV‐N1‐F 5′‐GAC CCC AAA ATC AGC GAA AT‐3′; and nCoV‐N1‐R 5′‐TCT GGT TAC TGC CAG TTG AAT CTG‐3′ was used and ACTIN was included as the reference gene for normalization. A Ct value of 40 was used as a limit of detection by RT‐PCR.

### 4.6 Immuno‐electron microscopy

For immunogold labeling, 5 μL specimen was absorbed to glow discharged carbon‐coated Formvar grids for 2 min. After rinsing with PBS containing 0.05% bovine serum albumin, grids were incubated with rabbit anti‐Spike primary antibody (Sino Biological; Catalog # 40150‐R007) or goat anti‐ApoE primary antibody (Millipore Sigma; Catalog # 178479) at 1:400 dilution for 15 min. After washing, grids were incubated with a 10 nm gold‐particle‐conjugated secondary antibody (Ted Pella; rabbit anti‐goat; Catalog # 17410−5 or goat‐anti‐rabbit, Catalog # 17010−5) at 1:50 dilution for 15 min. Finally, immunolabeled samples were negatively stained with 1% uranyl acetate for 20 s. Electron microscopy images were taken on an FEI Tecnai 12 transmission electron microscope equipped with a Gatan OneView CMOS camera.

### 4.7 Immunohistochemistry

Immunohistochemistry (IHC) analysis was performed following the protocols we described previously.^42^ Briefly, the antibodies specific for Spike (1:100; Sino Biological; Catalog # 40150‐R007), LDLR (1:25; R&D System; Catalog # AF2148), and ACE2 (1:100; Sigma; Catalog # AMAB91262) were used for IHC staining.

### 4.8 Antibody

The antibody specific for Spike (1:200; Sino Biological; Catalog # 40150‐R007) and the antibody specific for Vimentin (1:200; Millipore Sigma; Catalog # CBL202) were used for immunostaining. For immunostaining, images of infected cells were taken in a blinded manner before cell counting, five image fields per group were counted for quantification. Goat anti‐ApoE antibody (2 μg per IP; Millipore Sigma; Catalog # 178479), rabbit anti‐Spike antibody (2 μg per IP; Sino Biological; Catalog # 40150‐R007) were used for IP. Goat anti‐ApoE antibody (Millipore Sigma; Catalog # 178479), rabbit anti‐Spike antibody (1:1000; Sino Biological; Catalog # 40150‐R007), guinea anti‐Spike antibody (1:10,000; BEI Resources, NIAID, NIH; Catalog # NR‐10361), mouse anti‐Spike antibody (1:1000; Gene Tex; Catalog # GTX632604), mouse anti‐FLAG antibody (1:1000; SIGMA; Catalog # F1804), mouse anti‐GAPDH antibody (1:5000; Santa Cruz Biotechnology; Catalog # sc‐47724), rabbit anti‐NRP1 antibody (1:500; Millipore Sigma; Catalog # AB9600), goat anti‐LDLR antibody (1:500; R&D System; Catalog # AF2148), and goat anti‐ACE2 antibody (1:2000; R&D System; Catalog # AF933) were used for Western blot. The following reagents were obtained through BEI Resources, NIAID, NIH: Polyclonal Anti‐SARS Coronavirus (antiserum, Guinea Pig), NR‐10361.

## AUTHOR CONTRIBUTIONS

Y. S., V. A., P. F., and Q. C. designed the experiments and interpreted the results. Q. C., A. J., G. G., C. Q., and Y. Z. performed SARS‐CoV‐2 infection and sample collection. Q. C. and M. Z. performed experiments using astrocytes. Q. C. and C. W. performed experiments using NPC. G. S. prepared ApoE overexpression constructs. Q. C., W. L., T. Z., and C. P. performed WB analyses. W. L. and L. F. performed IHC staining analysis. Z. L. performed immuno‐electron microscopy analysis. A. A. and B. G. performed ALI experiment. Q. C. and Y. S. prepared the manuscript with inputs from other authors. All authors have read and approved the final manuscript.

## CONFLICT OF INTEREST STATEMENT

City of Hope has filed a U.S. Provisional Patent with Application No. 63/506,983 covering aspects of the technologies disclosed in this manuscript. The authors declare no other competing interests.

## ETHICS STATEMENT

There are no animal and clinical experiments involved in this study. The SARS‐CoV‐2 pseudovirus or SARS‐CoV‐2 virus study was performed under IBC protocols approved by the IBC committee of City of Hope, UCLA and USC. Human lung tissues without identifiers were obtained from Banner Sun Health Research Institute under IRB protocol 20120821 approved by the Western Institutional Review Board.

## Supporting information

Supporting Information

## Data Availability

All data reported in this paper will be available from the correspondence upon reasonable request.
