# Supplementary material for: The Apolipoprotein E neutralizing antibody inhibits SARS‐CoV‐2 infection by blocking cellular entry of lipoviral particles
Source: MedComm (2020). 2023 Oct 10;4(5):e400. doi: 10.1002/mco2.400 (PMC10563865; doi:10.1002/mco2.400)
Supplement: Supplementary file 1 — Supporting Information [file MCO2-4-e400-s001.pdf]

Supplementary Information for

The ApoE Neutralizing Antibody Inhibits SARS-CoV-2 Infection by Blocking Cellular Entry of Lipoviral Particles

Qi Cui[1], Arjit Vijey Jeyachandran[2], Gustavo Garcia Jr.[2], Chao Qin[3], Yu Zhou[3], Mingzi Zhang[1], Cheng Wang[1], Guihua Sun[1], Wei Liu[1], Tao Zhou[1], Lizhao Feng[1], Chance Palmer[1], Zhuo Li[4], Adam Aziz[5,6,7,8,9], Brigitte N. Gomperts[5,6,7,8,9], Pinghui Feng[3], Vaithilingaraja Arumugaswami[2,8], Yanhong Shi[1,*]

[*]Correspondence should be addressed to Y.S.

email: yshi@coh.org

This file includes:

Supplementary Figures 1 to 4

Supplemental Figures and Legends

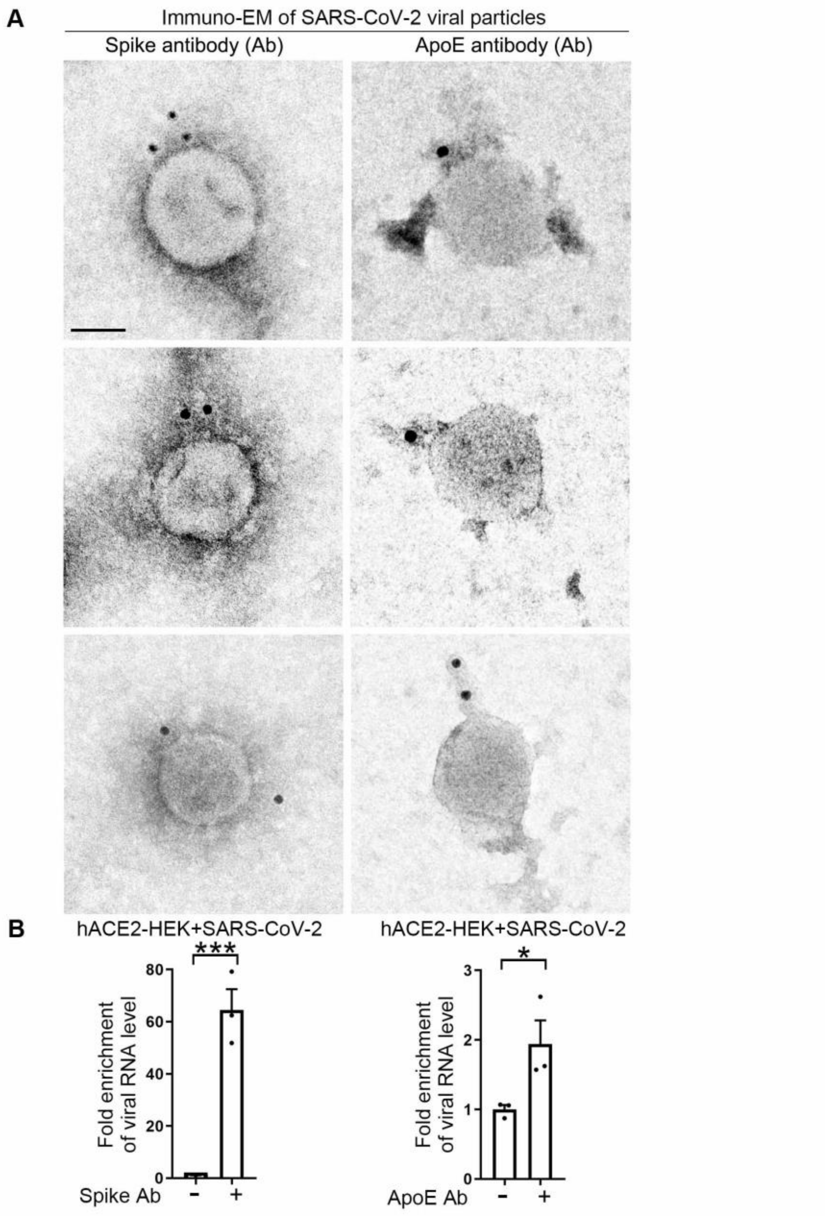

Figure S1 ApoE is part of the lipoviral particles of SARS-CoV-2. (**A**) Immuno-electron microscopy (EM) images showing the presence of Spike and ApoE on SARS-CoV-2 lipoviral particles by immunostaining SARS-CoV-2 viral particles with the Spike- or ApoE-specific antibody (Ab). The spike+ or ApoE+ signals are shown in black dots. Scale bar: 50 nm. (**B**) RT-PCR analysis showing fold enrichment of viral RNA level by immunoprecipitation of SARS-CoV-2 viral supernatant with the spike or ApoE-specific antibody (Ab). The corresponding IgG (- spike Ab or - ApoE Ab) was included as the negative control. The fold enrichment of viral RNA level in the pull-down by the spike or ApoE antibody was relative to the viral RNA level in the pull down by the control IgG. n=3 experimental replicates. Error bars are SE of the mean. *p < 0.05 and ***p < 0.001 by Student's t test.

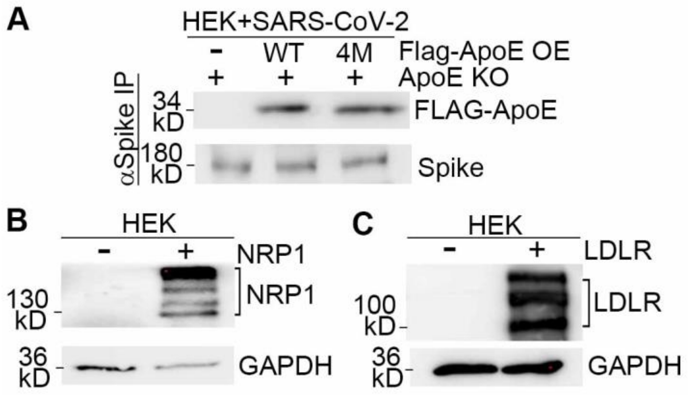

Figure S2 Overexpression of ApoE and LDLR. (**A**) Western blot analysis of Spike and FLAG-ApoE in SARS-CoV-2 viral particles collected from SARS-CoV-2-infected ApoE KO HEK cells that overexpressed the WT or the 4M mutant ApoE, followed by immunoprecipitation (IP) using a Spike antibody. (**B, C**) Western blot analysis of NRP1 (B) or LDLR (C) in HEK cells with or without NRP1 or LDLR overexpression.

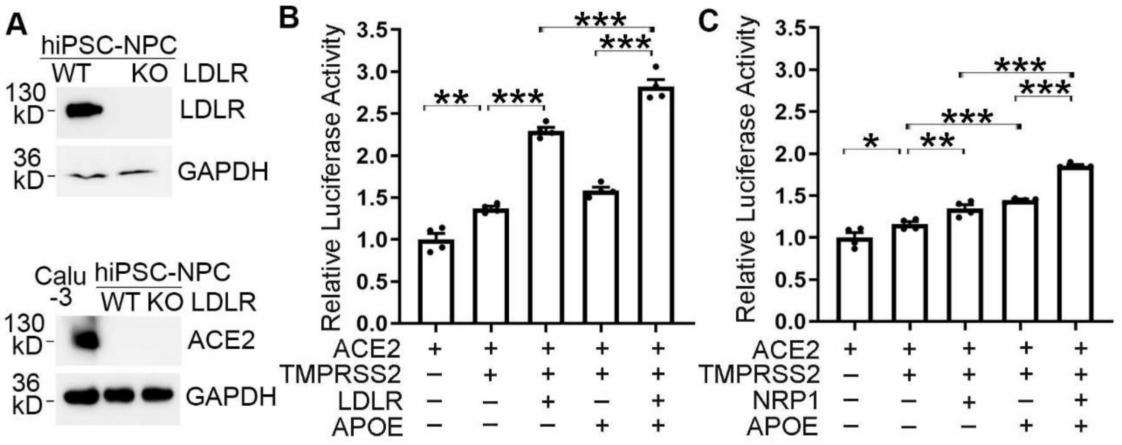

Figure S3 LDLR facilitates SARS-CoV-2 infection. (**A**) Western blot of LDLR (upper panel) and ACE2 (lower panel) in hiPSC-derived neural progenitor cells (NPC) with or without LDLR knockout (KO). (**B**) Treatment of HEK cells with or without ACE2, TMPRSS2, LDLR, and/or ApoE overexpression by pseudotyped SARS-CoV-2. The cellular entry of the pseudotyped virus was evaluated using a luciferase reporter assay. n=4 experimental replicates. (**C**) Treatment of HEK cells with or without ACE2, TMPRSS2, NRP1 and/or ApoE overexpression by pseudotyped SARS-CoV-2. The cellular entry of the pseudotyped virus was evaluated by a luciferase reporter assay. n=4 experimental replicates. Error bars are SE of the mean. *$p < 0.05$, **$p < 0.01$, and ***$p < 0.001$ by One-way ANOVA followed by Dunnett's multiple comparisons test for panels B and C.

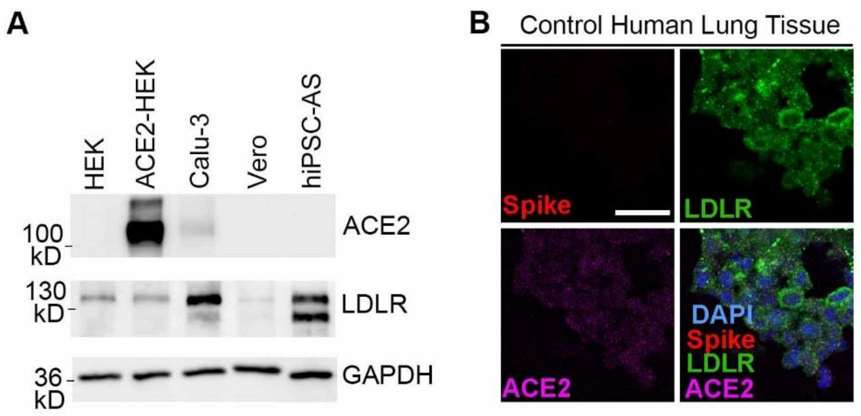

Figure S4 LDLR expression in cells and SARS-CoV-2 infected tissues. (**A**) Western blot of ACE2 or LDLR in HEK, Calu-3, Vero, or iPSC-derived astrocytes (AS). (**B**) Immunohistochemical staining for Spike, LDLR, and ACE2 on human lung tissues from control individual. Scale bar: 20 µm.
